# Consensus on gut feelings in general practice

**DOI:** 10.1186/1471-2296-10-66

**Published:** 2009-09-17

**Authors:** Erik Stolper, Paul Van Royen, Margje Van de Wiel, Marloes Van Bokhoven, Paul Houben, Trudy Van der Weijden, Geert Jan Dinant

**Affiliations:** 1 School for Public Health and Primary Care (CAPHRI), Department of General Practice, Maastricht University, Maastricht, The Netherlands; 2 Department of General Practice, University of Antwerp, Antwerp, Belgium; 3 Department of Work and Social Psychology, Maastricht, Maastricht University, The Netherlands

## Abstract

**Background:**

General practitioners sometimes base clinical decisions on gut feelings alone, even though there is little evidence of their diagnostic and prognostic value in daily practice. Research to validate the determinants and to assess the test properties of gut feelings requires precise and valid descriptions of gut feelings in general practice which can be used as a reliable measuring instrument. Research question: Can we obtain consensus on descriptions of two types of gut feelings: a sense of alarm and a sense of reassurance?

**Methods:**

Qualitative research including a Delphi consensus procedure with a heterogeneous sample of 27 Dutch and Belgian GPs or ex-GPs involved in academic educational or research programmes.

**Results:**

After four rounds, we found 70% or greater agreement on seven of the eleven proposed statements. A "sense of alarm" is defined as an uneasy feeling perceived by a GP as he/she is concerned about a possible adverse outcome, even though specific indications are lacking: *There's something wrong here*. This activates the diagnostic process by stimulating the GP to formulate and weigh up working hypotheses that might involve a serious outcome. A "sense of alarm" means that, if possible, the GP needs to initiate specific management to prevent serious health problems. A "sense of reassurance" is defined as a secure feeling perceived by a GP about the further management and course of a patient's problem, even though the doctor may not be certain about the diagnosis: *Everything fits in*.

**Conclusion:**

The sense of alarm and the sense of reassurance are well-defined concepts. These descriptions enable us to operationalise the concept of gut feelings in further research.

## Background

Uncertainty and unpredictability are common phenomena in general practice. [1] Unexplained complaints and ill-defined syndromes together form the group of uncertain diagnoses and uncertainty remains a characteristic part of medical life. [2-4] Although gut feelings can play a role in dealing with this diagnostic and prognostic uncertainty, [5-7] studies about the validity of gut feelings are lacking.

A qualitative study using four focus groups of 28 GPs in the Netherlands distinguished two types of gut feelings: a sense of alarm and a sense of reassurance. [8] Gut feelings are based on the recognition of a pattern that agrees or disagrees with the expected pattern for an individual patient or for a clinical picture, sometimes without a specific diagnosis. Although GPs are not always aware of their sense of reassurance, a sense of alarm alerts GPs and starts or re-starts the process of diagnostic reasoning: something does not fit in. This sense of alarm makes a GP feel uneasy and restless until the reason has been found. Sometimes there is a lack of objective arguments and the sense of unease remains. Three elements are important in defining a sense of alarm: the feeling that there seems to be something wrong without the doctor having objective arguments, a distrust of the situation because of uncertainty about the prognosis of the complaints, and the need for some kind of intervention to prevent serious health problems. When GPs experience a sense of reassurance, they are sure about the prognosis and therapy, even in the absence of a diagnosis. Gut feelings thus act as a compass in situations of uncertainty. To follow-up on the four focus groups and to operationalise this concept in further research and educational programmes, we organized a consensus procedure among opinion leaders and experts in general practice to explore if sufficient agreement could be reached on precise and valid descriptions of both types of gut feeling.

## Methods

**Figure 1:**
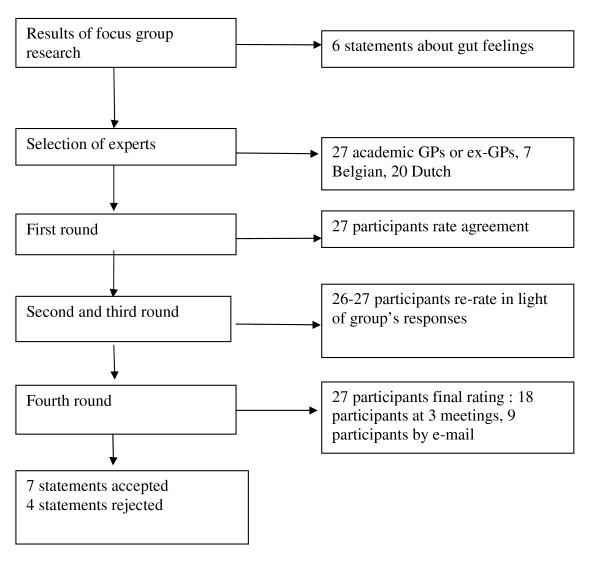
**flowchart Delphi consensus procedure about gut feelings**.

A modified Delphi consensus procedure was used combining several convential postal rounds and one face-to-face group session (see Figure 1: flowchart Delphi consensus procedure about gut feelings). The aim of such a procedure, named after the famous Delphic oracle, is to determine the extent to which experts agree about a given issue[9,10] This anonymous process was organised via a series of structured documents, including a number of statements, sent by post or e-mail to all participants, inviting them to rate their agreement on a scale from 1 (total disagreement) to 9 (total agreement). We encouraged the participants to explain their ratings, at least in the case of a rating lower than 7, by adding comments on the statements. Afterwards, these ratings and comments were used by two researchers (ES, PvR) to adjust the statements. After each round, the ratings and comments were used by two researchers (ES, PvR) to accept a statement or to adjust or reject the statements (if there was less than 70% agreement with a rating of 7 or higher). This phase of the Delphi technique involved an important qualitative component of considering, deliberating, weighing arguments and comments, thinking it over and finally deciding together (ES, PvR) to change a statement using different wording, another phrase or more fitting expression. However, the participants' rating afterwards played a decisive role in assessing whether an adaptation was an improvement or not. This whole process was checked by the co-authors. After each round, the ratings and comments were summarised and incorporated in a new version of the document. The participants then re-rated their agreement with each statement, with the possibility to change their rating in view of the group's response. The rounds were repeated till consensus was reached or seemed impossible. Not until the fourth round were the participants informed about the results of our previous focus-group study into gut feelings in order to prevent any bias by this information. The fourth round involved three meetings with 5-7 participants each, where they discussed the adjusted statements in group and rated them individually for the last time without giving written comments. The nine absent participants rated the statements afterwards by e-mail. Consensus in favour of a statement was defined as 70% or more agreement with a rating of 7 or higher.

We started with six statements, which were selected by the project group (i.e. the authors) and were based on the results of our previous focus-group study (see Appendix 1: statements submitted to Delphi participants). Next, we purposively sampled well-known opinion leaders and experts in general practice in the Netherlands and Belgium, who were working at universities in educational or research programmes, since the consensus statements had to be suitable for educational and research studies on the topic. We approached 30 colleagues by phone, 27 of whom accepted our invitation and received written information about the procedure. Because no patients were involved and GPs were only asked about their opinion and perception, no ethical permission was required. During the Delphi-procedure, all statements and comments were formulated in Dutch; afterwards all statements were translated into English and back-translated, in order to check for the right wording.

## Results

Four rounds were needed to reach consensus. During the entire process, eleven statements were presented to the participants, the six original ones and five new ones that emerged from the comments. Seven statements were accepted and four rejected (see Appendix 2: accepted statements; Appendix 3: rejected statements). There was a high level of response, and a large number of comments were given per round (see Table 1: number of comments (n), consensus (%) per round and final result in terms of agreement or disagreement with the proposed statements). In the first round, the participants were invited to add their own statements about gut feelings. Two supplementary statements were then incorporated in the next three rounds (statements 7 and 8). One statement was a comprehensive definition of gut feelings, while the other expressed the dynamic character of gut feelings. However, it proved to be impossible to get sufficient agreement about one definition that included all aspects of both types of gut feeling, despite several adjustments.

**Table 1:** Number of comments (n), consensus (%) per round and final result in terms of agreement or disagreement with the proposed statements.

**Statement**	**Round 1**		**Round 2**		**Round 3**		**Round 4**	**Result**	**In round**
	**n**	**%**	**n**	**%**	**n**	**%**	**%**		

1	26	37						Rejected	1

1a			18	76	4	100		Accepted	3

1b			16	69				Rejected	2

2	26	33	12	73	9	76	89	Accepted	4

3	19	59	10	92	3	100		Accepted	3

4	22	59	17	58	17	58	89	Accepted	4

5	19	44	12	92	8	88	85	Accepted	4

6	20	33	14	77	10	62		Rejected	3

7			24	33	18	42		Rejected	3

8			19	31	19	39	74	Accepted	4

9			18	50	11	77	78	Accepted	4

Although uncertainty emerged as a key word from the focus groups, it was difficult to keep it in, due to disagreement about the first statement presented to the Delphi panel. In this statement we had tried to unify the two types of gut feeling in one phrase about the degree of uncertainty. Although the significance of uncertainty as a central element in the concept of gut feelings had resulted from our previous research, it seemed too theoretical to be used to summarise the two types of gut feeling in one statement in the first round. Therefore, we split up the first statement (into 1a and 1b) in the second round and offered all statements relating to the sense of alarm separately from those relating to the sense of reassurance. Although some GPs commented in the last round that they would have preferred the notion of uncertainty to be included, it is still implied in statements 1a and 3 as "uneasy feeling", "worries" and "there is something wrong here" and in statement 5 as "secure feeling" and "uncertain about the diagnosis" (see Appendix 2: accepted statements).

In the second round we added another statement (9) about the process of gut feelings, based on the comments of the participants. Statement 6 was accepted in round 2, but further explanation, intended to reach a higher degree of consensus, confused the participants and we therefore withdrew this statement. Furthermore, statement 5 already comprised all elements of the sense of reassurance.

In the course of this Delphi procedure, the statements gradually became more focused. After three rounds, we had already reached sufficient consensus about several statements, but the consensus even increased after the wording was further adjusted. In the end, it was not difficult to distinguish between accepted and rejected statements.

## Discussion

We reached consensus on a broadly based and precise description of the two types of gut feeling: a sense of alarm and a sense of reassurance. The key elements in the results of our earlier focus group study were confirmed and transformed into clear, practical descriptions that could be used by doctors participating in general practice research. A sense of alarm is defined as an uneasy feeling perceived by a GP as he/she is concerned about a possible adverse outcome, even though specific indications are lacking: *There's something wrong here*. This activates the diagnostic process by stimulating the GP to formulate and weigh up working hypotheses that might involve a serious outcome. A sense of alarm means that, if possible, the GP initiates specific management to prevent serious health problems. A sense of reassurance is defined as a secure feeling perceived by a GP about the further management and course of a patient's problem, even though he/she may not be certain about the diagnosis: *Everything fits in*.

We distinguished four interrelated dimensions in the accepted statements: the meanings of the sense of alarm and the sense or reassurance (statements 1a, 3 and 5), the vague and uneasy prognostic feeling lacking clear causes (statements 1a and 3) and the consequences of the sense of alarm (statements 2 and 4). And the statements 8 and 9 express that this is not a steady state: a sense of alarm is sometimes replaced by a sense of reassurance during the encounter and vice versa.

The two types of gut feelings are not each other's mirror images. The essential element of the sense of alarm is the lack of a diagnosis whereas a clear diagnosis can reassure a GP, even though it may actually be an unfavourable diagnosis for the patient.

Several participants associated gut feelings with feelings of empathy towards the patient but the topic of our research was the significance of GPs' gut feelings in the diagnostic process, rather than the GPs' empathy, which has no diagnostic value. Empathy comes into play after the diagnosis has been established, for instance in the case of an unfavourable diagnosis, when the doctor has to initiate treatment and/or define a management plan, whereas the gut feelings we wish to study are used in the diagnostic process itself. In the second round, the participants were therefore asked to use this basic assumption as a starting point. Several colleagues expressed their disagreement with this decision in their ratings.

The Delphi consensus technique has been used widely in health care research [9,10] and its validity and trustworthiness have been the topic of many debates. However, we followed the guidelines for the use of this consensus technique[11] and the transparency of the way we dealt with the comments and ratings and how we adjusted or rejected statements may have improved the validity and reliability of the consensus achieved. Although this Delphi consensus procedure only included 27 participants, they were all well-known experts from 8 universities in two countries with wide experience both as GPs and researchers or medical educators. Their representativeness for general practice and their power to implement the findings may contribute to the generalizability of the results. Furthermore, the consensus is in line with the focus group results. The procedure started with statements based on our earlier focus group research, without the participants being aware of this. The validity of the focus group results was checked by comparing it with the results achieved by the consensus procedure, a process commonly referred to as triangulation. [12,13] Compared with the results of the focus groups, the descriptions of both types of gut feeling have now been improved and have become more precise and complete than before.

Another possible weakness of our study is what is known as regression to the mean: participants are inclined to adjust their opinions during the process of finding consensus[14] Nevertheless, the degree of agreement reached about seven statements was high and four statements were not accepted despite several adjustments.

## Conclusion

We conclude that the sense of alarm and the sense of reassurance are well-defined concepts and the descriptions resulting from the Delphi procedure enable us to operationalise the concept of gut feelings in further research into the validity of this "compass" as well as educational programmes.

## Competing interests

The authors declare that they have no competing interests.

## Authors' contributions

ES and PVR carried out the Delphi consensus procedure and drafted the manuscript. MVdW, MVB, PH, TVdW and GJD participated in the design of the study, assisted in carrying out the procedure and helped to draft the manuscript. All authors read and approved the final manuscript.

## Appendix 1: statements submitted to Delphi participants

• Statement 1: A GP's 'sense of reassurance or alarm' is mostly related to their degree of certainty about the prognosis of the complaints.

• Statement 2: The 'sense of reassurance or alarm' has very little to do with formulating working hypotheses or establishing diagnoses.

• Statement 3: A 'sense of alarm' implies that a GP is worried about a patient's health status, even though he or she has as yet no objective argument for this; it is a sense of 'there's something wrong here'.

• Statement 4: A 'sense of alarm' means that some form of intervention seems necessary to prevent imminent serious health problems.

• Statement 5: A 'sense of reassurance' means that a GP feels secure about the prognosis, even though there are no objective arguments for this: everything fits in.

• Statement 6: A 'sense of reassurance' implies that a GP feels secure about whether and what therapy needs to be started.

## Appendix 2: accepted statements

• Statement 1a: A 'sense of alarm' means that a GP perceives an uneasy feeling as he/she is concerned about a possible adverse outcome.

• Statement 3: A 'sense of alarm' implies that a GP worries about a patient's health status, even though he/she has found no specific indications yet; it is a sense of 'there's something wrong here'.

• Statement 2: A 'sense of alarm' activates the diagnostic process by stimulating a GP to formulate and weigh up working hypotheses that might involve a serious outcome.

• Statement 4: A 'sense of alarm' means that, if possible, the GP needs to initiate specific management to prevent serious health problems

• Statement 9: A 'sense of alarm' will decrease as the diagnosis and the right management become clearer.

• Statement 5: A 'sense of reassurance' means that a GP feels secure about the further management and course of a patient's problem, even though he/she may not be certain about the diagnosis: everything fits in.

• Statement 8: The 'sense of reassurance' and the 'sense of alarm' constitute a dynamic element in a GP's diagnostic process.

## Appendix 3: rejected statements

• Statement 1: A GP's 'sense of reassurance or alarm' is mostly related to their degree of certainty about the prognosis of the complaints.

• Statement 1b: A 'sense of reassurance' means that a GP feels at ease as he or she is confident about the further approach and outcome.

• Statement 6: A 'sense of reassurance' implies that a GP has a clear idea whether a particular therapy would be useful and needs to be started.

• Statement 7: A 'sense of alarm' is a sensation/feeling that a doctor is unable to express in specific terms and that is prompted by data from medical history-taking and/or examination of a patient. It helps the doctor in taking further diagnostic and therapeutic decisions in order to prevent a potentially serious outcome for the patient.

## Pre-publication history

The pre-publication history for this paper can be accessed here:


